# Beyond the Colon: Acute Hemolysis Associated With Etrasimod Therapy

**DOI:** 10.7759/cureus.111344

**Published:** 2026-06-23

**Authors:** Sohaib Ahmed, Saud Khan, Eric Vargas Carranza, Syed Usman Mumtaz, Michael Delzoppo

**Affiliations:** 1 Internal Medicine, University of Central Florida, Orlando, USA; 2 Internal Medicine, UCF/HCA, Orlando, USA; 3 Internal Medicine, HCA Healthcare, Kissimmee, USA

**Keywords:** autoimmune hemolytic anemia (aiha), drug induced immune hemolytic anemia, drug-related adverse reactions, etrasimod, sphingosine-1-phosphate receptor modulators, ulcerative colitis (uc)

## Abstract

Etrasimod is an oral sphingosine 1-phosphate (S1P) receptor modulator used in the treatment of moderately to severely active ulcerative colitis (UC). Although its safety profile has been described in clinical studies, hematologic complications remain incompletely characterized. We report the case of a 31-year-old man with well-controlled UC who developed acute hemolytic anemia approximately two months after initiation of etrasimod therapy. Evaluation revealed macrocytic anemia with marked reticulocytosis, schistocytes on peripheral smear, elevated ferritin, and a positive direct antiglobulin test (IgG-positive, complement-negative), consistent with immune-mediated hemolysis. Extensive evaluation excluded infectious, metabolic, and hereditary causes. Discontinuation of etrasimod and transfusion support resulted in incomplete improvement; subsequent high-dose corticosteroid therapy led to recovery of hemoglobin levels and resolution of hemolysis. To our knowledge, this is the first reported case of immune-mediated hemolytic anemia associated with etrasimod therapy. Awareness of this rare but potentially serious adverse event is important as the clinical use of S1P receptor modulators continues to expand.

## Introduction

Ulcerative colitis (UC) is a chronic inflammatory bowel disease characterized by relapsing and remitting mucosal inflammation limited to the colon. Over the past decade, management of UC has expanded beyond conventional aminosalicylates, corticosteroids, and thiopurines to include targeted biologic and small-molecule therapies [1-4]. These agents aim to modulate specific immune pathways involved in intestinal inflammation, improving disease control while limiting systemic toxicity.

Etrasimod is a selective oral sphingosine 1-phosphate (S1P) receptor modulator that preferentially targets S1P receptor subtypes 1, 4, and 5 [3-4]. By inducing partial sequestration of lymphocytes within lymphoid tissues, etrasimod reduces circulating lymphocyte counts and attenuates intestinal inflammation. Phase 2 and 3 clinical trials have demonstrated its efficacy as both induction and maintenance therapy in moderately to severely active UC, leading to its approval by the U.S. Food and Drug Administration (FDA) in 2023 [1,3].

Reported adverse effects of etrasimod include headache, dizziness, nausea, hypertension, bradycardia, and transient elevations in liver enzymes [1-4]. Mild anemia has been observed in clinical trials; however, hemolytic anemia has not been reported [1-3]. Drug-induced immune hemolytic anemia (DIIHA) is an exceptionally rare but potentially life-threatening condition, with an estimated incidence of approximately one per one million individuals [5]. We present the first reported case of acute immune-mediated hemolytic anemia temporally associated with etrasimod therapy in a patient with UC.

This article was previously presented as a poster at the 2025 American College of Gastroenterology Annual Scientific Meeting on October 26, 2025.

## Case presentation

A 31-year-old man with a history of UC and hypertension presented to the hospital after routine pre-endoscopy laboratory testing revealed macrocytic anemia with a hemoglobin of 6.8 g/dL. His UC had been well controlled, and his medical therapy had recently been transitioned from tofacitinib to etrasimod approximately two months before presentation. His only other medication was amlodipine for hypertension.

He denied alcohol use, recent travel, new medications, over-the-counter supplements, or illicit drug use. He reported progressive fatigue over the preceding month but denied dyspnea, chest pain, palpitations, bleeding, or jaundice. He endorsed a single self-limited episode of diarrhea two weeks prior but denied symptoms suggestive of a UC flare. There was no history of fever, night sweats, unintentional weight loss, or family history of malignancy, hematologic disorders, or autoimmune disease. On examination, he was hemodynamically stable and afebrile. Physical examination was notable only for conjunctival pallor; there was no scleral icterus, lymphadenopathy, hepatosplenomegaly, or stigmata of chronic liver disease.

Initial laboratory studies demonstrated macrocytic anemia with a markedly elevated reticulocyte count of 22%, consistent with a robust marrow response (Table 1). Liver function tests, renal function, thyroid studies, urinalysis, prothrombin time/international normalized ratio, partial thromboplastin time, and fibrinogen levels were within normal limits. D-dimer was mildly elevated. Iron studies revealed elevated ferritin levels. Peripheral blood smear showed schistocytes, polychromatophilia, and occasional teardrop cells, suggestive of ongoing hemolysis. Glucose-6-phosphate dehydrogenase (G6PD) testing was negative. Further evaluation for secondary causes of hemolysis was unrevealing. Viral serologies, including hepatitis A, B, C, and HIV, were negative. Immunoglobulin levels were within normal limits, without evidence of monoclonal gammopathy. Cold agglutinin testing was negative. A direct antiglobulin test (DAT) was positive for IgG and negative for complement, supporting an immune-mediated hemolytic process (Table 2). Other relevant labs are presented in Table 3.

**Table 1:** Pertinent abnormal laboratory findings at initial presentation LDH: lactate dehydrogenase; MCV: mean corpuscular volume; DAT: direct antiglobulin test; IgG: immunoglobulin G

Test	Result	Reference range
Hemoglobin	6.8 g/dL	13.5-17.5 g/dL
Reticulocyte count	22%	0.5-2.5%
LDH	640 U/L	140-280 U/L
Haptoglobin	Low	30-200 mg/dL
MCV	108 fL	80-100 fL
DAT (IgG)	Positive	Negative
Total bilirubin	Elevated	0.1-1.2 mg/dL
Ferritin	Elevated	30-400 ng/ml

**Table 2:** Relevant immunology labs DAT: direct antiglobulin test; IgG: immunoglobulin G; G6PD: glucose-6-phosphate dehydrogenase

Immunology labs	
DAT (IgG)	Positive
DAT (C3)	Negative
Cold agglutinin	Negative
G6PD	Normal
Immunoglobulins	Normal

**Table 3:** Other relevant labs PT: prothrombin time; PTT: partial thromboplastin time

Other labs	
D-dimer	Mildly elevated
Liver function	Normal
Renal function	Normal
PT/PTT	Normal
Viral panel	All negative

**Figure 1:**
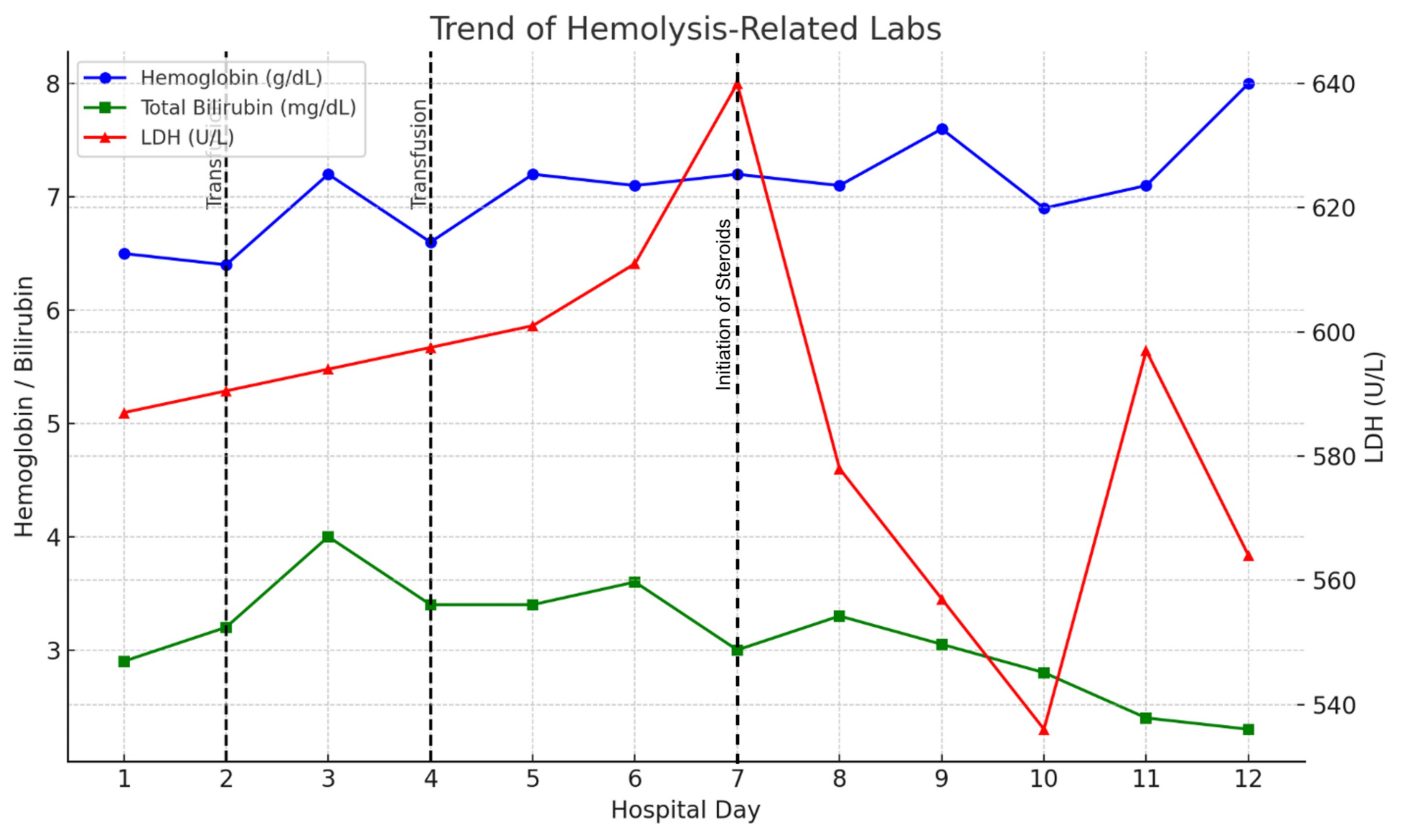
Trend of hemolysis-related labs Etrasimod held: day 0. Blood transfusions given: days 2 and 4. Oral prednisone 100 mg therapy initiated: day 7 LDH: lactate dehydrogenase

Given concerns for drug-induced hemolytic anemia, etrasimod was discontinued. The patient received two units of packed red blood cells for symptomatic anemia. Despite discontinuation of the suspected offending agent, serial laboratory testing demonstrated persistent biochemical evidence of hemolysis. Following multidisciplinary discussion with hematology, high-dose corticosteroid therapy with prednisone 100 mg daily was initiated on hospital day seven. Subsequently, hemoglobin levels stabilized and began to improve, with resolution of hemolytic markers (Figure 1).

The patient was discharged on oral prednisone 100 mg daily, with plans to continue this regimen for two weeks as an outpatient. Follow-up with hematology was arranged for ongoing monitoring and subsequent corticosteroid tapering. A follow-up colonoscopy performed in the outpatient setting after clinical stabilization showed no evidence of active UC flare. The patient continued to improve clinically and hematologically on steroid therapy.

## Discussion

DIIHA is a rare but serious adverse drug reaction, accounting for a small fraction of all cases of hemolytic anemia [6-8]. Several pathogenic mechanisms of DIIHA have been described, including the hapten (drug adsorption) mechanism, immune complex formation, and true autoantibody induction [7-8]. In the latter mechanism, the offending drug triggers the production of autoantibodies directed against red blood cell antigens, resulting in hemolysis that may persist even after drug discontinuation [7-9].

The clinical and laboratory findings in our patient were most consistent with drug-induced autoantibody formation. The presence of a DAT positive for IgG in the absence of complement supports a warm autoimmune hemolytic anemia-like process, a pattern more characteristic of drug-induced autoantibody production than immune complex-mediated hemolysis [7-8]. Furthermore, the persistence of hemolysis despite initial drug discontinuation and the subsequent favorable response to corticosteroid therapy with oral prednisone 100 mg daily are consistent with an immune-mediated process driven by autoantibodies [7-9]. In contrast, alternative mechanisms such as immune complex formation are considered less likely given the absence of complement positivity on DAT and the overall clinical presentation.

The temporal relationship between etrasimod initiation and the onset of hemolytic anemia, together with an extensive evaluation that excluded alternative infectious, metabolic, and hereditary etiologies, further strengthens the likelihood of a causal association. Notably, clinical improvement occurred following withdrawal of etrasimod and initiation of immunosuppressive therapy. To further assess causality, the Naranjo Adverse Drug Reaction Probability Scale was applied and yielded a score of 6, classifying the relationship between etrasimod exposure and the development of hemolytic anemia as a “probable” adverse drug reaction. While causality cannot be definitively established from a single case, the cumulative clinical, laboratory, and temporal evidence strongly supports etrasimod-induced autoantibody-mediated hemolysis as the most likely explanation for this patient's presentation.

Etrasimod’s immunomodulatory effects are mediated via selective S1P receptor modulation, leading to altered lymphocyte trafficking and immune regulation [3-4]. While the precise mechanism by which etrasimod may precipitate immune hemolysis remains unclear, immune dysregulation and loss of self-tolerance may contribute. Clinical trials and long-term extension studies have reported anemia as an adverse event; however, hemolytic anemia has not been previously described in association with etrasimod therapy [1-3].

An alternative consideration in this case is autoimmune hemolytic anemia as an extraintestinal manifestation of UC itself. However, several features of this presentation make this explanation less likely. In the largest available series, autoimmune hemolytic anemia demonstrated a near-exclusive association with colonic inflammatory bowel disease, with 93% of affected patients carrying a diagnosis of UC, supporting the hypothesis that colonic inflammation serves as the immunologic source of cross-reactive autoantibody production. Importantly, the investigators found that the clinical course of autoimmune hemolytic anemia and inflammatory bowel disease activity ran largely in parallel, with hemolysis worsening during periods of active colitis and improving as intestinal inflammation was controlled [10]. In contrast, our patient’s UC was clinically and endoscopically quiescent, making UC-related hemolysis less likely. The resolution of anemia following corticosteroid therapy further supports an immune-mediated drug reaction rather than an extraintestinal manifestation of UC.

As the use of novel small-molecule therapies continues to expand, recognition of rare and unexpected adverse events becomes increasingly important. Post-marketing surveillance, along with case reporting, remains essential for defining the full safety profile of newly approved agents such as etrasimod [1,6].

## Conclusions

This case report highlights a previously unreported association between etrasimod and immune-mediated hemolytic anemia. Clinicians should consider drug-induced immune hemolysis in patients who develop unexplained anemia after the initiation of novel immunomodulatory therapies, even in the absence of active underlying inflammatory disease. Prompt recognition of the condition, discontinuation of the suspected offending agent, and initiation of appropriate immunosuppressive therapy, including high-dose prednisone (100 mg oral daily) as used in this case, may be critical for successful treatment and recovery.
